# Auxin Coordinates Achene and Receptacle Development During Fruit Initiation in *Fragaria vesca*

**DOI:** 10.3389/fpls.2022.929831

**Published:** 2022-07-07

**Authors:** Yunhe Tian, Wei Xin, Juncheng Lin, Jun Ma, Jun He, Xuhui Wang, Tongda Xu, Wenxin Tang

**Affiliations:** ^1^College of Horticulture, Fujian Agriculture and Forestry University, Fuzhou, China; ^2^Plant Synthetic Biology Center, Horticulture Biology and Metabolic Center, Haixia Institute of Science and Technology, Fujian Agriculture and Forestry University, Fuzhou, China; ^3^College of Ecology and Resources Engineering, Wuyi University, Wuyishan, China

**Keywords:** fruit set initiation, pollination, auxin, achene, receptacle, phytohormone

## Abstract

In strawberries, fruit set is considered as the transition from the quiescent ovary to a rapidly growing fruit. Auxin, which is produced from the fertilized ovule in the achenes, plays a key role in promoting the enlargement of receptacles. However, detailed regulatory mechanisms for fruit set and the mutual regulation between achenes and receptacles are largely unknown. In this study, we found that pollination promoted fruit development (both achene and receptacle), which could be stimulated by exogenous auxin treatment. Interestingly, auxin was highly accumulated in achenes, but not in receptacles, after pollination. Further transcriptome analysis showed that only a small portion of the differentially expressed genes induced by pollination overlapped with those by exogenous auxin treatment. Auxin, but not pollination, was able to activate the expression of growth-related genes, especially in receptacles, which resulted in fast growth. Meanwhile, those genes involved in the pathways of other hormones, such as GA and cytokinin, were also regulated by exogenous auxin treatment, but not pollination. This suggested that pollination was not able to activate auxin responses in receptacles but produced auxin in fertilized achenes, and then auxin might be able to transport or transduce from achenes to receptacles and promote fast fruit growth at the early stage of fruit initiation. Our work revealed a potential coordination between achenes and receptacles during fruit set, and auxin might be a key coordinator.

## Introduction

Fruit growth origins from floral organogenesis and goes through fruit set, development, and ripening process, which produces edible fruits (Gillaspy et al., 1993; Giovannoni, 2004; Handa et al., 2012). Fruit set is the first and critical stage for fruit growth (Gu et al., 1998; Fuentes and Vivian-Smith, 2009). In the majority of plants, fruit set is dependent on fertilization; thus, flowers without pollination and fertilization will senesce and fall off. Phytohormones play crucial roles during fruit set after pollination and fertilization (Ohad et al., 1996; Viviansmith et al., 2001; Shinozaki et al., 2020). Exogenous hormone treatments can induce fruit set (parthenocarpy) without pollination in many plant species, such as *Arabidopsis* (Roeder and Yanofsky, 2006; Fuentes and Vivian-Smith, 2009; Carbonell-Bejerano et al., 2011), cucumber (Su et al., 2021), tomato (Gorguet et al., 2005; Serrani et al., 2007; Jong et al., 2009), and strawberry (Kang et al., 2013). These findings have been applied in both horticulture and agriculture in order to produce seedless and high-quality fruits. The regulatory mechanisms of phytohormones in fruit set attracted intense interest during past years (Sharif et al., 2022).

Auxin is one of the most important phytohormones that regulate fruit set in plants. After fertilization, the auxin level dramatically increases in fruit (Gorguet et al., 2005; Serrani et al., 2008) and is required for further regulation of fruit set initiation (Fuentes and Vivian-Smith, 2009). Auxin promotes the transcriptional activity of *auxin response factors* (*ARFs*) by inducing the degradation of the AUX/IAA transcription repressors (Gray et al., 2001). Previous studies show that both *ARFs* and *AUX/IAAs* are required for the regulation of fruit set (Fuentes and Vivian-Smith, 2009; Jong et al., 2009). Knock-down of *AtARF8* in *Arabidopsis* (Goetz et al., 2006), *SlARF7* in tomato (Jong et al., 2009), or *SmARF8* in eggplant (Du et al., 2016) leads to fruit set initiation without pollination and fertilization. Both loss-of-function (Zhang et al., 2007) and antisense (Wang et al., 2005) mutants of *SlIAA9* cause fast fruit growth independent of pollination and fertilization in tomatoes.

Gibberellin (GA) is another hormone critical for fruit set (Serrani et al., 2008; Alabadi et al., 2009). DELLA proteins, key components in GA signaling, act as repressors of GA responses (Sun, 2011). In *Arabidopsis*, the quadruple mutant of *DELLAs* (*RGA, GA insensitive, RGL1*, and *RGL2*) can initiate fruit set without fertilization (Dorcey et al., 2009; Fuentes et al., 2012). In tomatoes, functional defects of *SlDELLAs* can induce fruit development without fertilization (Marti et al., 2007; Carrera et al., 2012; Nir et al., 2017). Fruit growth initiation is an energy-consuming process, and the gibberellin-signaling cascade regulates central carbon metabolism by upregulating metabolic enzyme capacities at both transcriptional and posttranscriptional levels (Shinozaki et al., 2020). Furthermore, evidence reveals that auxin induces GA biosynthesis during fruit set. Auxin undergoes crosstalk with GA through the protein–protein interaction between ARF and DELLA during fruit set (Serrani et al., 2008; Hu et al., 2018; Zhou et al., 2021), proves that auxin and GA are critical for fruit set initiation, but studies on the regulation between seeds and fleshy fruits are still limited.

Strawberry has a unique fruit structure containing the receptacle (fleshy fruit) and the achene (seed), and achenes attach on the surface of receptacles. It offers a great system to investigate the relationship between seeds and fruits during fruit set initiation (Hollender et al., 2012; Kang et al., 2013; Cappelletti et al., 2015; Hartl et al., 2017). In *Fragaria vesca* (*F. vesca*), both phenotype and transcriptome analyses reveal the crucial roles of both auxin and GA in the enlargement of fruits (Kang et al., 2013; Liao et al., 2018). By comparing gene expression patterns, the genes required for the biosynthesis of both auxin and GA (*FvYUCs, FvGA20ox*, and *FvGA3ox*) are expressed in achenes, while the genes required for both perception (*FvTIR1* and *FvGID1*) and signaling (*FvIAAs*/*FvARFs* and *FvRGA1*) are highly expressed in the receptacles (Kang et al., 2013). This finding suggests that auxin and GA might be first synthesized and accumulated in the fertilized ovaries and then be transported to receptacles and promote fruit enlargement (Kang et al., 2013). Thus, receptacles fail to expand after removing the attached achenes (Nitsch, 1950; Ishibashi et al., 2017). The mutation of *FvRGA1* leads to the parthenocarpy of strawberry (Zhou et al., 2021). These findings elucidate the critical roles of auxin and GA in the fruit development of strawberry. However, the regulatory mechanism of fruit set by auxin, especially at early stages, is still quite unclear.

## Materials and Methods

### Plant Materials and Growth Conditions

The diploid *Fragaria vesca*. Yellow wander 5AF7 was used in this study (Slovin et al., 2009). Seedlings were grown in the growth room under 16-h light at a temperature of 23 ± 2°C and 55% humidity. The flower opening stage was defined as 0 day after anthesis (DAA). Pollination was manually operated at 0 DAA to obtain pollinated fruits (Shinozaki et al., 2020). The emasculation of fruits that were used for chemical treatments was performed using tweezers. Developmental patterns of the fruits were studied from−2 DAA to 6 DAA. The fruits were frozen immediately in liquid nitrogen, and then achenes and receptacles were separated under frozen conditions.

### Hormone Measurements

For extraction, 900 μL ethyl acetate and 100 μL of 50 ng/mL [^13^C_6_]-IAA (OlChemIm), 50 ng/mL [^15^C_4_]-trans zeatin (OlChemIm), 50 ng/mL ^2^H_6_-ABA (OlChemIm), and 50 ng/mL [^2^H_4_]-SA (OlChemIm) isotope internal standard were added to each sample (100 mg). The samples were vortexed and sonicated for 20 min at 4°C and centrifuged at 12,000 g for 10 min at 4°C. The supernatant was transferred into a new 2-mL centrifugal tube and then was centrifuged to dry. The precipitation was redissolved with 200 μL of precooled 70% methanol. After filtration through a 0.22-μm PVDF filter, the solution was taken in a sample bottle and then analyzed by UPLC-QqQ MS (ACQUITY TQD).

### Hormone Treatment

Hormone treatment was performed at 0 DAA, and the flowers were immersed in the corresponding treatment solutions for 10 s. For fruits used for RNA-seq and phenotype analysis, hormone treatment was applied only one time at 0 DAA. The final treatment concentrations were 500 μM for NAA and GA3, and 100 μM for PAC, with 0.1% ethanol and 0.01% Tween 20 (Liao et al., 2018).

### Fruit and Achene Development Analysis

About 15–20 fruits from at least five plants were collected for each treatment with three biological replicates. Totally, 60 individual achenes were collected from each treatment at the corresponding stages. The size of fruits and achenes were measured.

### RNA Isolation and Gene Expression Analysis

Polysaccharide and polyphenolics-rich RNAprep Pure kit (Tiangen) was used for RNA extraction in this study. DNA was removed by the on-column DNase digestion with RNase-Free DNase I (Tiangen). After quality inspection, 0.5–1 μg of total RNA was used to synthesize cDNA by using a cDNA Synthesis SuperMix kit (TransGen). Using *FvACTIN* as an internal control, the quality and quantity of cDNA were analyzed. Gene expression analysis was performed by real-time quantitative PCR (qRT-PCR) analyses by using the CFX96TMReal-Time System with the SYBR^®^ Green Supermix (Bio-Rad). The primer sequences used for the qRT-PCR, and accession numbers of the analyzed genes are shown in Supplementary Table 3.

### Generation of Transcriptome Libraries, Functional Annotation, and DEG Identification

To generate the transcriptome library, 1–2 ug of total RNA for each sample was sent to Novogene for RNA sequencing. After interrupting RNA by the NEB Next^®^Ultra™II RNA Library Prep Kit for Illumina^®^, sequencing was performed with the Illumina NovaSeq 6000 (150-bp paired-end reads). 6G sequencing depth was performed for each sample. Fastp 0.22.0 was used to qualify the raw data based on length > 18 bp and mass > 30. The clean data were mapped to *Fragaria vesca* Genome v4.0.a2 (www.rosaceae.org/species/fragaria_vesca/genome_v4.0.a2) by STAR 2.7.6a. When both read pairs aligned uniquely, they were used toward gene read counts. Then, featureCounts v2.0.1 (Yang et al., 2014; Radke et al., 2022) was used to count the non-strand-specific reads for gene exon features based on the Genome v4.0.a2 gene model. Gene functional annotation was performed based on Fragaria_vesca_v4.0.a2 protein and Fragaria_vesca_v4.0.a2 transcript annotation provided in Rosaceae website (https://www.rosaceae.org/species/fragaria/all).

The differentially expressed genes (DEGs) were screened out using DESeq2 between the two samples for achenes and receptacles based on |log2(fold change)| > 1.5 and *P-*value < 0.05. Adjusted through the Benjamini–Hochberg method, six groups of DEGs were identified.

### PCoA and Violin Analysis

Principal co-ordinates analysis (PCoA) was performed to analyze the overall expression profiles in different samples. Violin analysis was performed to understand the overall gene expression level distribution. PCoA and violin analysis were performed based on the whole transcriptome data using OmicShare, a free online platform for data analysis (https://www.omicshare.com).

### Venn and Heatmap Analyses

Venn diagram was created based on DEGs (https://www.omicstudio.cn/tool6). Heatmaps of target genes were generated by OmicStudio tools (https://www.omicstudio.cn/tool4) and Hiplot (https://hiplot.com.cn/basic/heatmap). The cluster method was ward.D2.

### Functional Enrichment Analysis and Result Display

GO enrichment analysis (*p*-value < 0.05) of the DEGs was performed using OmicShare, an online platform for data analysis (www.omicshare.com/tools). The GO function was annotated based on the database of Fvesca_v4.0.a2_genes2Go, which was downloaded from Rosaceae website (https://www.rosaceae.org/species/fragaria_vesca/genome_v4.0.a2). For the similar GO terms with the same genes, only one terms were retained based on *P-*value < 0.05 and *Q*-value < 0.05. The enrichment results showed in the bar plot, bubble gradient, and circular diagram were generated by GraphPad Prism 8 (https://www.Graphpad.Com/scientific-software) and Omicshare (https://www.omicshare.com/~tools/Home/Soft).

### Quantification and Statistical Analysis

Statistical analysis was performed by using GraphPad Prism 8. Statistical significances were determined using *t* tests and/or two-way ANOVA.

## Results

### Both Auxin and Pollination Promoted Receptacle and Achene Enlargement During Fruit Set

**Figure 1:**
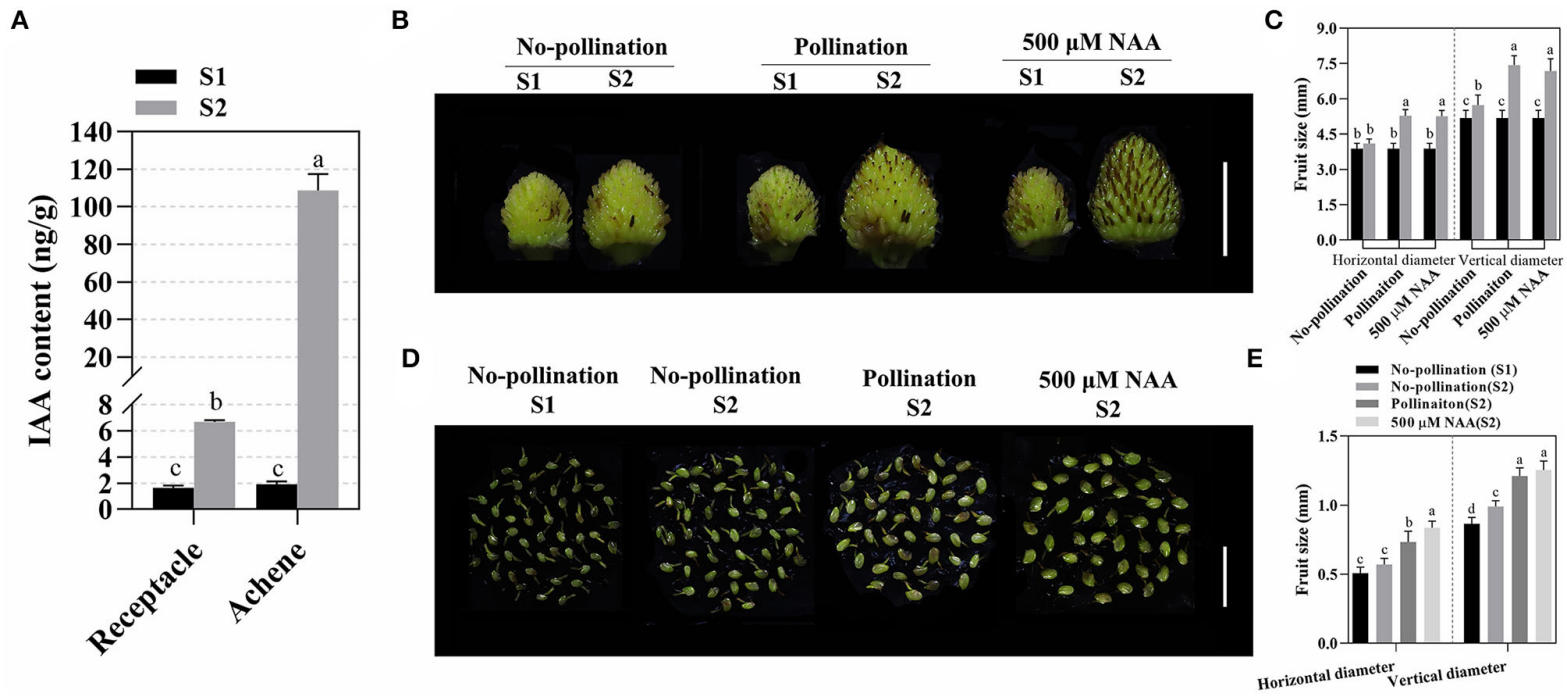
Auxin accumulated in fruits and promoted fruit development. **(A)** IAA was highly accumulated in receptacles and achenes during fruit set. Data are represented as means ± SD (*n* = 3, one-way ANOVA). About 15–20 fruits were used for each replicate, and the achenes and receptacles were sampled separately. S1 and S2 indicate stage 1 and stage 2, respectively. Statistical analysis shows the significant difference between the groups (*p* < 0.05, one-way ANOVA). **(B)** Fruit development after pollination or auxin treatment. Scale bar = 0.5 cm. **(C)** Quantification of vertical and horizontal diameters for fruits after pollination or auxin treatment. Error bar represents SD, *n* = 15–20. Statistical analysis showed the significant difference between the groups (*p* < 0.05, one-way ANOVA). **(D)** Achene growth after pollination or auxin treatments. Scale bar = 0.5 cm. **(E)** Quantification of achene size in different conditions. Error bars represent SD, *n* = 60 achenes from 15 to 20 fruits. Statistical analysis indicated the significant difference between the groups (*p* < 0.05, one-way ANOVA).

Pollination and fertilization are essential for fruit set initiation in *F. vesca*. In this process, the phytohormones accumulated in different tissues of fruit which were required for fruit set initiation (Nitsch, 1950; Kang et al., 2013). By liquid mass spectrometry, we evaluated the levels of phytohormones, including auxin (IAA), abscisic acid (ABA), salicylic acid (SA), and cytokinin (trans-zeatin and t-zeatin), in fruit (achene and receptacle) at stage 1 (S1) and stage 2 (S2). A lower IAA content was identified in both receptacles and achenes before pollination. After fruit initiation, IAA was increased both in receptacles and achenes, while a higher IAA content was found in achenes (about 57 times) than in receptacles (about four times; Figure 1A), suggesting that auxin might be involved in the development of both achenes and receptacles during fruit set. Meanwhile, other hormones, including t-zeatin, ABA, and SA, were also analyzed. Similar to auxin, t-zeatin, an active cytokinin, increased dramatically in S2, especially in achenes (Supplementary Figure 1A). Different from IAA and t-zeatin, ABA decreased in receptacles but increased in achenes (Supplementary Figure 1B). SA levels decreased in both achenes and receptacles (Supplementary Figure 1C).

Based on the dynamic distribution pattern during fruit set, we speculated that auxin coordinates the fruit development after pollination. Thus, we tested the effect of auxin on early fruit set initiation. After pollination, the enlargements of fruit (both receptacle and achene) were induced at as early as 1 DAA (Supplementary Figure 2). At S2, exogenous auxin (500 μM NAA) significantly accelerated both horizontal and vertical expansion of fruit (both receptacle and achene), which was similar to the effect of pollination (Figures 1B–E). The results indicated that auxin is able to stimulate the effect of pollination, which promotes fruit development and induces parthenocarpy.

### Auxin Induced Rapid Fruit Initiation Partially Dependent on Gibberellin

**Figure 2:**
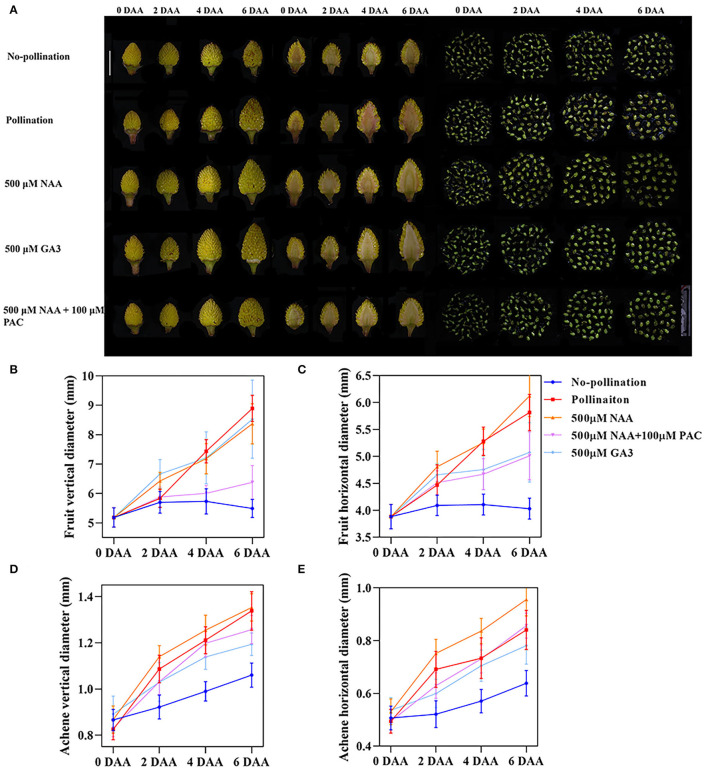
Auxin induced the rapid fruit initiation partially dependent on gibberellin. **(A)** Development of fruit and achene at different stages under varies growth conditions. Scale bar = 1 cm. **(B,C)** Vertical **(B)** and horizontal **(C)** diameters of fruit under varies treatments. Error bar represents SD, *n* = 15–20. **(D,E)** Vertical **(D)** and horizontal **(E)** diameters of achenes under various treatments. Error bar represents SD, *n* = 60.

Exogenous GA treatment has also been reported to induce parthenocarpy both in *F. vesca* (Kang et al., 2013) and other species (Fuentes and Vivian-Smith, 2009; Jong et al., 2009; Shinozaki et al., 2020). To test the relationship between auxin and GA on strawberry fruit growth, we applied both GA3 and PAC (paclobutrazol), a GA synthesis inhibitor, together with auxin on the emasculated flowers at 0 DAA (Figure 2A) and then examined fruit development from 0 DAA to 6 DAA by calculating both horizontal and vertical diameters (Figures 2B–E). Regarding receptacles, NAA strongly increased both horizontal and vertical diameters as pollination, while GA3 mainly promoted vertical enlargement. PAC inhibited the effect of NAA on both horizontal and vertical enlargement (Figures 2A–C). Our results together with previous research (Kang et al., 2013; Liao et al., 2018) suggested that auxin is a critical hormone in the regulation of fruit enlargement partially dependent on GA.

Fruit enlargement is caused by the enlargement of receptacles or achenes or both. By the longitudinal section, the achene growth rate was also measured in details. Compared to the unpollinated fruit, both NAA and GA3 strongly induced achene enlargement as pollination. As in receptacles, NAA preferentially induced the horizontal diameters, while GA3 induced vertical elongation of achenes (Figures 2D,E). Receptacles and achenes showed conspicuous enlargement before 2 DAA under both NAA and GA3 treatments, while the growth rate decreased from 2 DAA to 6 DAA compared with pollination (Figures 2B–E). The inhibition of receptacle growth by PAC was much more severe than that of the achene (Figure 2). The fruit vertical diameter was significantly inhibited by PAC (Figure 2B). The horizontal diameters of fruit treated by NAA + PAC were similar to those treated by GA3 (Figure 2C). The results suggested that auxin and GA have different functions on regulating the growth of achenes compared to those on receptacles. Auxin induced growth in both receptacles and achenes, and its function on receptacles was dependent on GA.

### Transcriptome Reprogramming by Pollination and Auxin During Fruit Set Initiation

**Figure 3:**
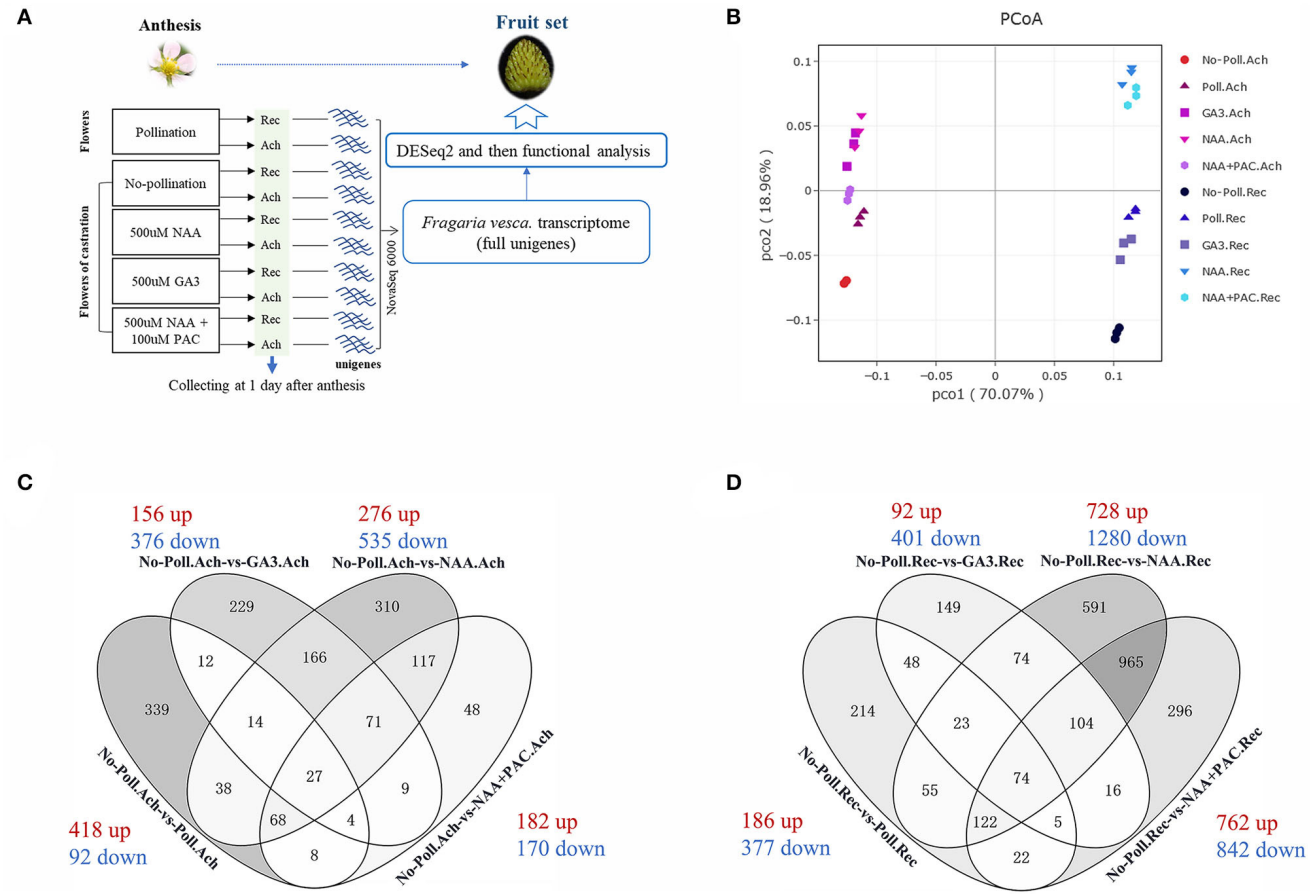
Global gene transcription in achenes and receptacles during fruit set initiation responding to auxin and pollination. **(A)** Experimental procedure for RNAseq analysis. Rec represents receptacle, and Ach represents achene. **(B)** PCoA based on gene expression. The whole transcription data were used, and X-ray and Y-ray show the distance between each sample. No-poll represents no-pollination. Poll represents pollination. **(C,D)** Overlapping set of the DEGs identified in the achenes **(C)** and receptacles **(D)** under different treatments. Numbers of the upregulated gene (red) and downregulated gene (blue) are shown for each corresponding groups. *P* < 0.05, |log2(fold change)| > 1.5.

To investigate the mechanism of pollination and auxin on regulating both receptacle and achene development during fruit set, RNA-seq analysis was performed in fruits with or without pollination, as well as fruits treated with NAA, GA3, NAA, and PAC (Figure 3A). Early at 1 DAA, pollination induced significant enlargement of both receptacles and achenes (Supplementary Figure 2). Therefore, we performed treatment at 0 DAA and collected the samples of receptacles and achenes separately at 1 DAA for RNA-seq (Figure 3A). After filtering out the low-quality data, 17–27 million reads about 5.74–8.14 G clean bases were collected from each sample. Then, the clean bases were used to map against the *F. vesca* reference genome (see Methods).

Global gene expression patterns were then analyzed by both PCoA (Figure 3B) and correlation dendrogram (Supplementary Figure 3B) after verifying the quality of the transcriptome data (Supplementary Figure 3A). The gene expression patterns in receptacles and achenes were clustered into two independent branches (Supplementary Figure 3B), indicating the different transcriptomes in receptacles and achenes. Compared to the emasculated fruits, pollination, auxin, and GA3 had obvious effects on gene expression in both achenes and receptacles. Especially, auxin induced the greatest change of gene expression patterns (Figure 3B). The pattern in the NAA + PAC-treated achenes is closer to that in the NAA-treated achenes (Figure 3B), but the patterns in receptacles were quite different, suggesting that auxin regulates receptacle development partially depending on GA.

To further analyze the gene expression profiles in achenes and receptacles under different treatments, different expression genes (DEGs) were screened out based on *p* < 0.05 and |log2(fold change)| > 1.5. In achenes, 510 DEGs in No-Poll.Ach-vs-poll.Ach, 532 DEGs in No-Poll.Ach-vs-GA3.Ach, 811 DEGs in No-Poll.Ach-vs-NAA.Ach, and 352 DEGs in No-Poll.Ach-vs-NAA+PAC.Ach were selected, respectively (Figure 3C). The majority of the identified DEGs were downregulated in GA3- or NAA-treated conditions. However, pollination induced most of upregulated DEGs. In achenes, 147 DEGs of NAA-treated samples overlapped with the samples with pollination, while 57 overlapped DEGs were found between GA treatment and pollination. Totally, 278 overlapped DEGs were found between NAA- and GA3-treated samples (Figure 3C).

In receptacles, 563 DEGs in No-poll.Rec-vs-Poll.Rec, 493 DEGs in No-Poll.Rec-vs-GA3.Rec, 2,008 DEGs in No-poll.Rec-vs-NAA.Rec, and 1,604 DEGs in No-poll.Rec-vs-NAA+PAC.Rec were selected, respectively (Figure 3D). Similar to the achene, the majority of the identified DEGs were downregulated in the GA3- or NAA-treated samples (Figures 3C,D). However, different from achenes, most of the identified DEGs were also downregulated in pollination. In receptacles, NAA and pollination induced 274 overlapped DEGs, and GA3 induced 150 overlapped DEGs with pollination. Overall, 275 overlapped DEGs were found in NAA- and GA3-treated samples (Figure 3D).

### Characterization of the DEGs Responding to Pollination and Auxin Treatment

**Figure 4:**
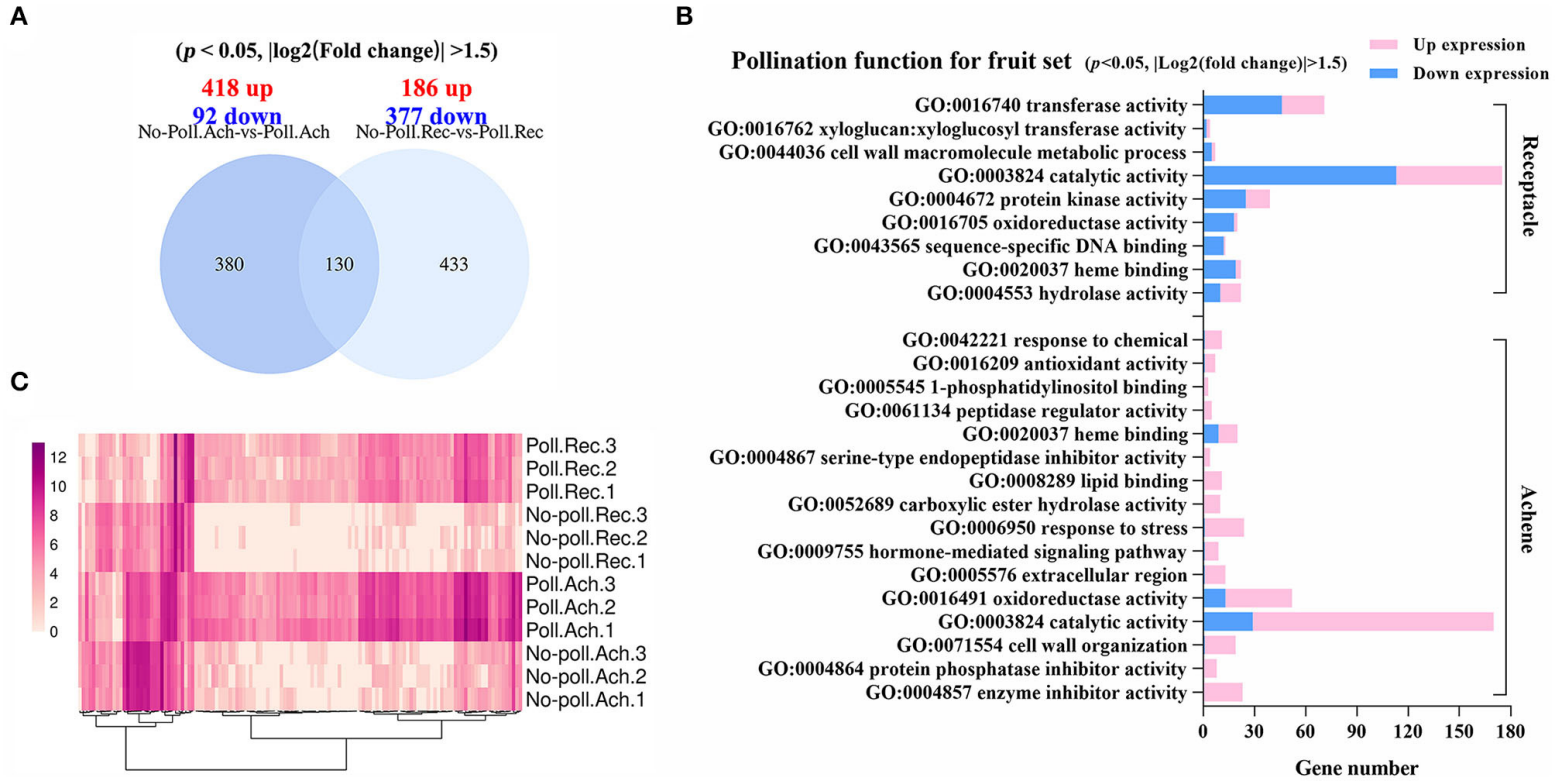
Gene regulation in response to pollination. **(A)** Venn shows the relationship for the DEGs identified in the pollinated achenes and receptacles (*p* < 0.05, |log2(fold change)| > 1.5). **(B)** Significantly enriched GO terms of the DEGs in achenes and receptacles, respectively. *P* < 0.05. **(C)** Heatmap analysis of the overlapping 130 DEGs. Expression values were converted into log2(value + 10^−6^).

The characterization of DEGs in different conditions was further performed (Figure 4). Pollination induced a number of DEGs in achenes (510) similar to receptacles (563), but the pattern was quite different (Figure 4A). Next, gene ontology (GO) functional enrichment analysis showed that different biological processes were regulated in receptacles and achenes by pollination.

In achenes, 16 GO terms were enriched for the 418 upregulated and 92 downregulated DEGs in hormone-mediated signaling pathways, serine-type endopeptidase inhibitor activity, protein phosphatase inhibitor activity, cell wall organization, etc. (Figures 4A,B). In receptacles, 186 upregulated and 377 downregulated DEGs were enriched into nine GO terms, including transferase activity, protein kinase activity, sequence-specific DNA binding, and xyloglucosyl transferase activity, suggesting pollination initiates the different developmental processes in achenes and receptacles (Figures 4A,B). The DEGs in achenes and receptacles shared the GO terms, including catalytic activity, heme binding, cell wall organization, and oxidoreductase activity (Figure 4B). Moreover, the 130 overlapped DEGs in both achenes and receptacles had a similar pattern after pollination (Figure 4C).

**Figure 5:**
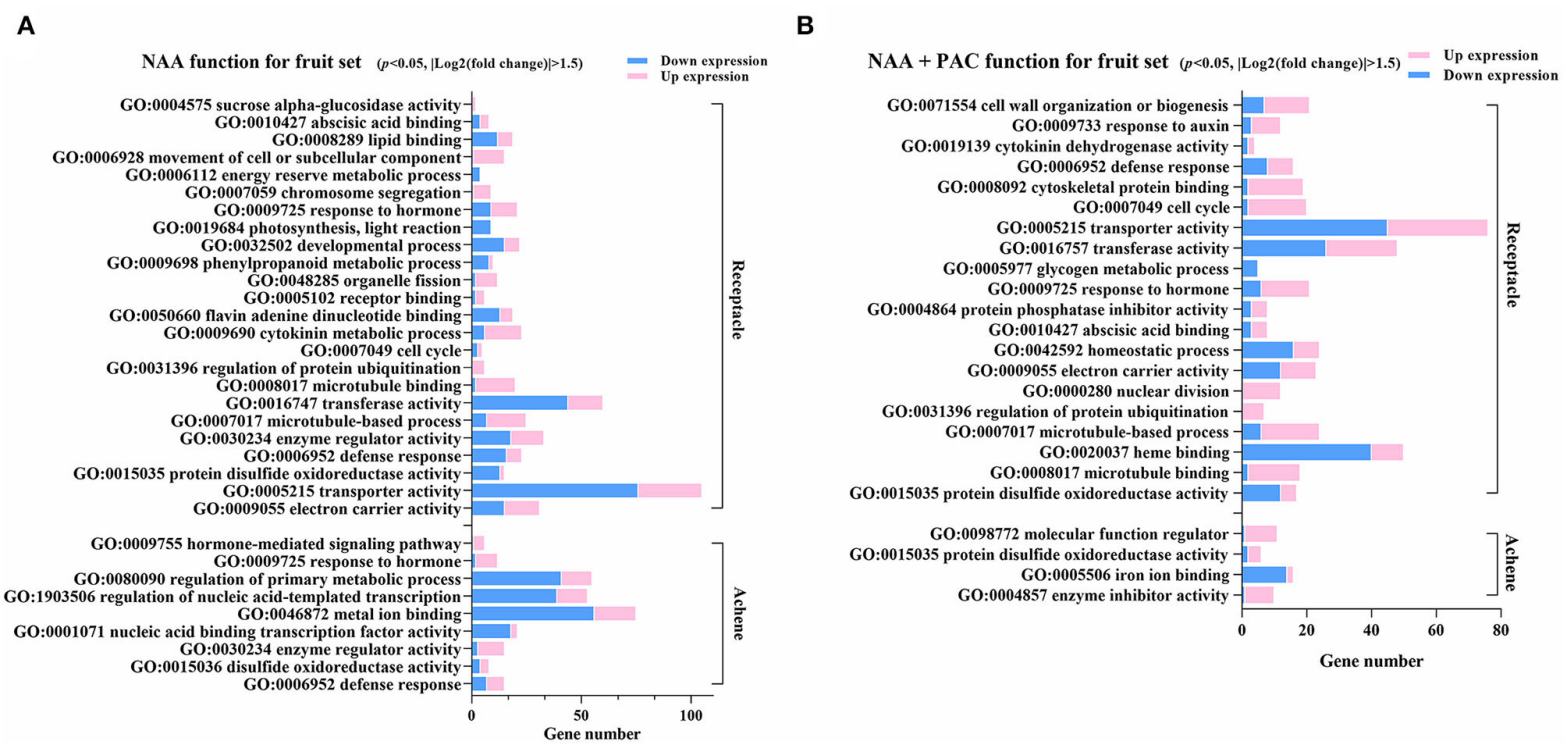
Enriched GO terms identified in a3henes and receptacles under NAA and NAA+PAC treatment. **(A)** Functional enrichment of GO terms of differential expressed genes responding to NAA treatment. DEGs for receptacles and achenes were analyzed, respectively. **(B)** GO terms for genes influenced by NAA + PAC. The up- or downregulated genes are noted in different colors. *P* < 0.05, |log2(fold change)| > 1.5.

DEGs identified in the NAA-treated receptacles were classified into 24 GO terms, much more than those in the NAA-treated achene, which had only nine GO terms (Figure 5A). In receptacles, NAA mainly activated the expression of genes involved in the movement of cell or subcellular component, chromosome segregation, organelle fission, cytokinin metabolic process, regulation of protein ubiquitination, and microtubule-based process, while genes related to transporter activity, transferase activity, and developmental processes were mainly downregulated. In achenes, NAA mainly activated the expression of genes related to hormone responses, signaling pathways, and enzyme regulator activity, while genes related to metal ion binding, regulation of nucleic acid-templated transcription, and regulation of primary metabolic processes were mainly downregulated. Receptacles and achenes only shared four GO terms (response to hormones, enzyme regulator activity, disulfide oxidoreductase activity, and defense response), again suggesting different responses in achenes and receptables after auxin treatment.

NAA treatment could induce DEGs that were clustered in much more GO terms than GA3 treatment or NAA + PAC treatment, suggesting the dominant role of NAA (Figure 5B, Supplementary Figures 4, 5). NAA and NAA+PAC shared several enriched GO terms, such as protein disulfide oxidoreductase activity, transporter activity, transferase activity, abscisic acid binding, cell cycle, cytokinin dehydrogenase activity, microtubule-based process, enzyme regulator activity, and defense response (Figure 5). The present results suggested that the effect of auxin on fruit development is both dependent and independent on GA.

### Distinctive Functions of Auxin and Pollination in Fruit Set Initiation

Both auxin and pollination played key roles during fruit initiation. Next, we investigated the relationship between auxin and pollination during fruit initiation by characterizing the DEGs identified in auxin treatment and pollination.

**Figure 6:**
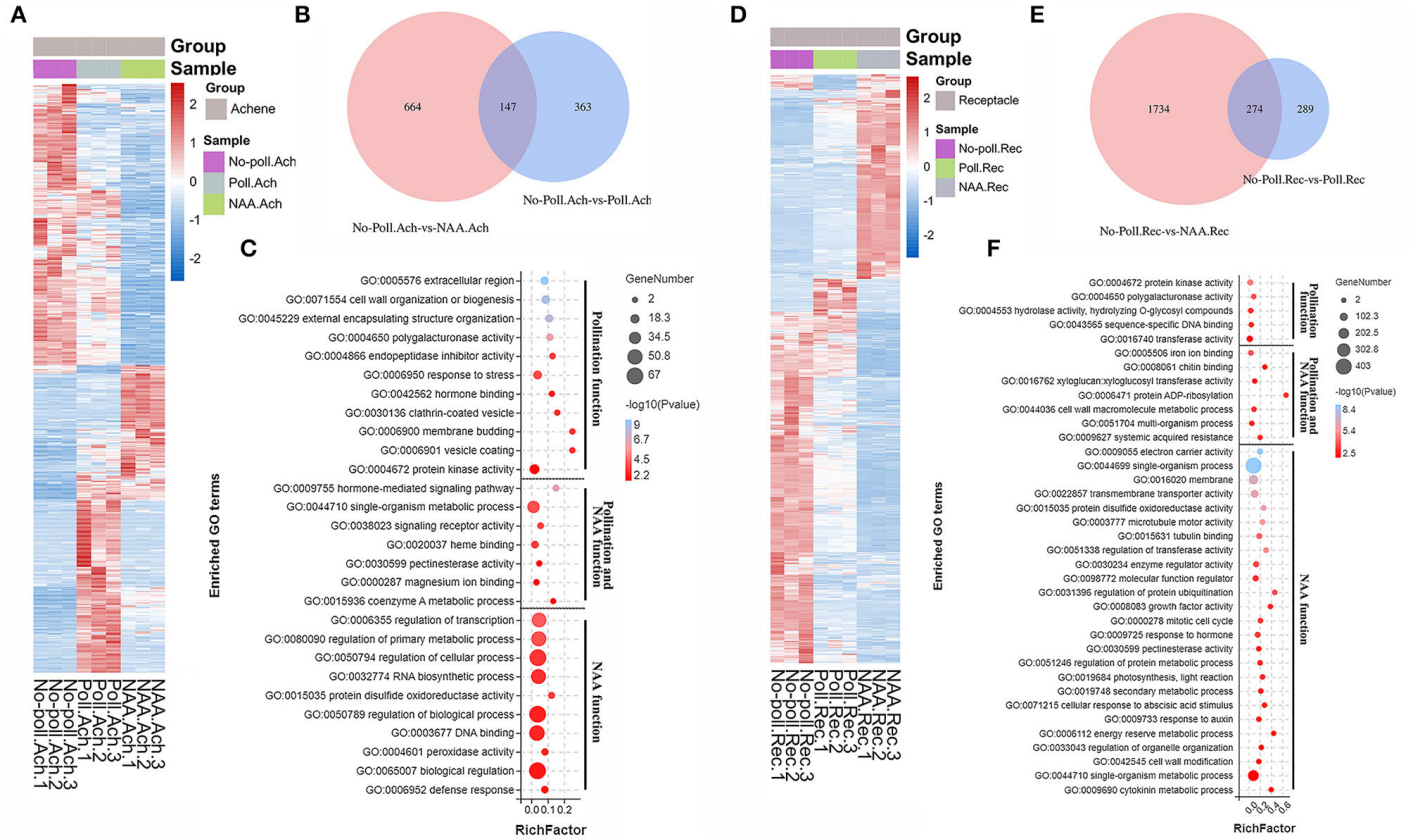
Gene regulation of auxin was much different in pollination during fruit set. Heatmap analysis of 1174 DEGs for achenes **(A)** and 2297 DEGs for receptacles **(D)** that were differentially regulated by pollination or NAA treatment. *P* < 0.05, |log2(fold change)| > 1.5. Expression values were converted into log2(value + 10^−6^). **(B,E)** Distribution diagrams of the DEGs identified in achenes and receptacles responding to pollination and NAA treatments. *P* < 0.05, |log2(fold change) >1.5|. **(C,F)** GO enrichment for the DEGs differentially regulated by pollination and NAA in achenes **(C)** and receptacles **(F)**. Legend in the right shows the enriched gene numbers and log10(P value). Function of pollination and NAA was analyzed separately. *P* < 0.05.

In achenes, a total of 1,174 DEGs were identified in either pollination or NAA-treated samples (Figures 6A–C). The expression patterns of these DEGs in the conditions of pollination and NAA were quite different (Figure 6A). The amount of DEGs responsive to NAA was larger than that responsive to pollination. Totally, 811 and 510 DEGs were identified as specific NAA-regulated or pollination-regulated genes, respectively, with only 147 overlapped genes (Figure 6B). The pollination-induced DEGs were classified into 18 GO terms, including cell wall organization or biogenesis, hormone binding, endopeptidase inhibitor activity, and protein kinase activity. The NAA-induced DEGs were classified into 17 GO terms, including regulation of transcription, regulation of primary metabolic processes, regulation of cellular processes, regulation biological processes, and DNA binding. Only seven GO terms overlapped in both conditions, including hormone-mediated signal pathway, single-organism metabolic processes, signal receptor activity, and pectinesterase activity (Figure 6C), suggesting the distinctive effects of auxin and pollination during achene development.

In receptacles, a total of 2297 DEGs were identified in either pollination or NAA-treated samples (Figures 6D–F). The heatmap analysis showed more DEGs under NAA-treated conditions than pollination (Figure 6D). In all, 2008 and 563 DEGs were identified as NAA and pollination-regulating genes, respectively, with only 274 overlapped DEGs (Figure 6E). The DEGs induced by pollination were classified into 12 GO terms, including protein kinase activity, polygalacturonase activity, and sequence-specific DNA binding. The DEG responses to NAA were classified into 32 GO terms, including single-organism process, transmembrane transporter activity, microtubule motor activity, regulation of protein ubiquitination, growth factor activity, mitotic cell cycle, secondary metabolic process, cellular response to auxin, abscisic acid, cytokinin, and cell wall modification. The overlapping DEGs belonged to seven GO terms, including iron ion binding, chitin binding, xyloglucosyl transferase activity, protein ADP-ribosylation, cell wall macromolecule metabolic process, multi-organism process, and systemic acquired resistance (Figure 6F). This again suggested the distinctive function of auxin and pollination in receptacle development.

Notably, a total of 340 classified DEGs were specifically responded to auxin and are not influenced by PAC treatment (Supplementary Figure 6). Heatmap analysis showed that these DEGs had similar expression patterns in achenes and receptacles with the obvious gene expression-level regulation elicited in receptacles (Supplementary Figure 6A). Based on functional enrichment analysis, these DEGs can be classified into 20 GO terms, including seven biological processes, 11 molecular functions, and two cellular components (Supplementary Figure 6B). Their functions were associated with transmembrane transporter activity, growth factor activity, receptor binding, cellular carbohydrate metabolic process, and so on. Notably, although PAC could inhibit the NAA effect on fruit enlargement (Figure 2), few DEGs between NAA and NAA+PAC were found in both achenes and receptacles (Supplementary Figure 5), indicating PAC might change the expression level, but not the type of genes.

### Auxin Activated More Genes Than Pollination in Receptacles

**Figure 7:**
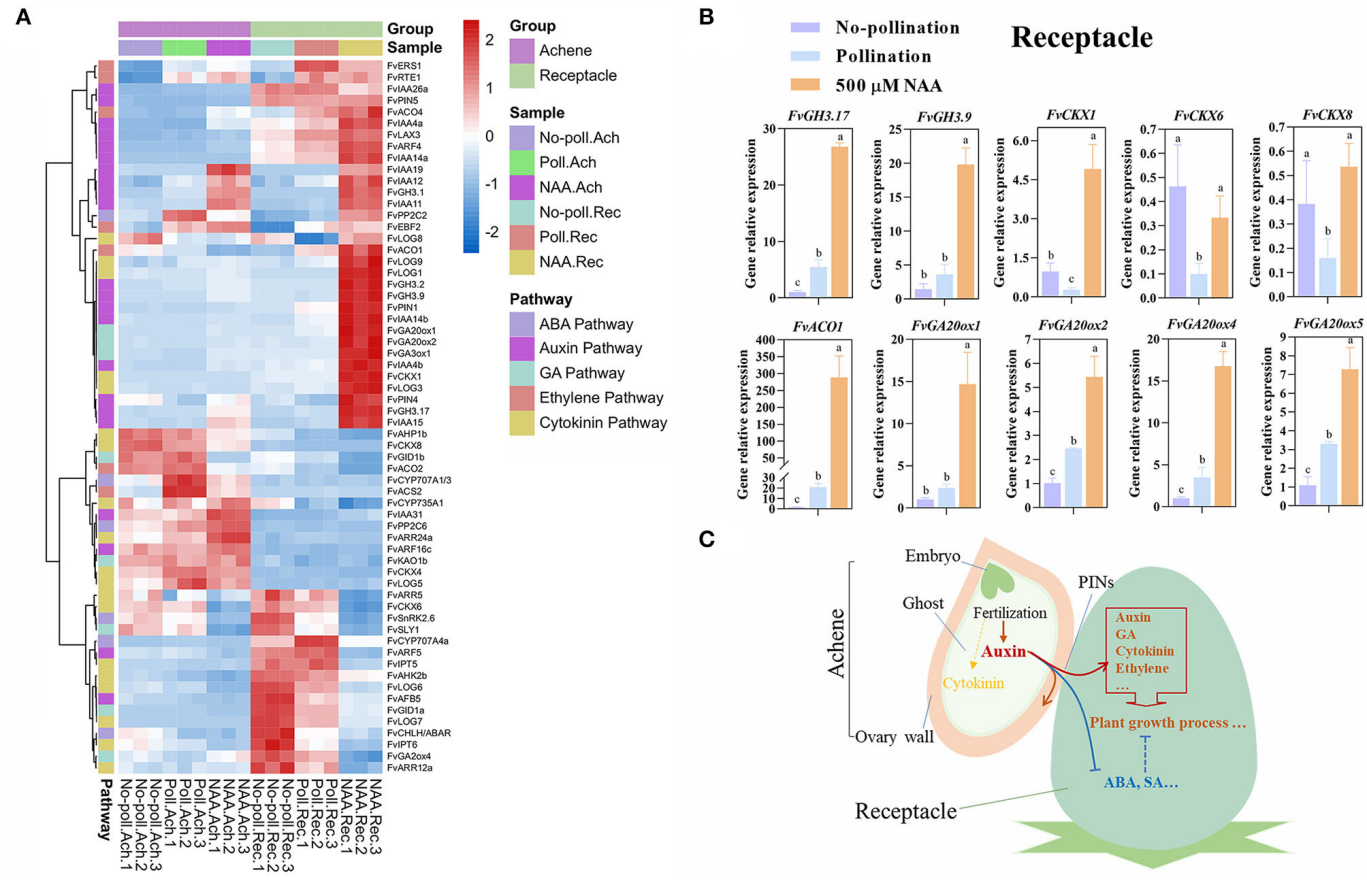
Characterization of auxin action during fruit set. **(A)** Expression patterns of hormone pathway genes. DEGs were classified through gene functional annotation and genes specially regulated by auxin in achenes or receptacles. For heatmap analysis, the expression values were transformed to log2(value+10^−6^). *P* < 0.05, |log2(fold change)| > 1.5. Hormone pathways are noted in the left line, and samples for five treatments and the samples belonging to achenes or receptacles were annotated. **(B)** Expression of several genes were verified in receptacles through qRT-PCR. Receptacles were treated with No-pollination, pollination, and 500 μM NAA for 1 day. Error bars represent SD of three independent replicates. Different characters represent the significant differences (*p* < 0.05, one-way ANOVA). **(C)** Predictive regulatory mechanisms for auxin in promoting fruit set.

Multiple phytohormones participate in fruit set, and their crosstalk ensures the precise regulation during this process (Sharif et al., 2022). Based on *p* < 0.05 and |log2(fold change)| > 1.5, 62 DEGs participating in the phytohormone pathway were screened out as pollination or auxin-responsive genes in achenes or receptacles (Figure 7A). Heatmap analysis showed that pollination has minor effects on the expression of these genes in both achenes and receptacles. Interestingly, auxin has much stronger effects on the expression of these genes in receptacles than in achenes (Supplementary Tables 1, 2).

In receptacles, in addition to 22 auxin-related genes, eight GA pathway genes were also regulated by auxin. Among them, three GA biosynthesis genes *FvGA20ox1, FvGA20ox2*, and *FvGA3ox1* were upregulated, while a GA catabolic gene of *FvGA2ox4* was downregulated. This further supports the conclusion that auxin induced the rapid fruit initiation partially depending on gibberellin. In addition, three biosynthesis genes (*FvLOGs*) and one catabolic gene (*FvCKX1*) in the cytokinin pathway, *FvPP2C2* in ABA, and five genes in the ethylene pathway were also upregulated. However, we also found *FvCHLH/ABAR* and *FvSnRK2.6* genes in the ABA pathway, two *FvIPTs* genes in the cytokinin pathway, and *salicylic acid-binding protein 2* were downregulated after auxin treatment, suggesting the complex roles of auxin on phytohormones (Figure 7A).

To confirm the effects of auxin and pollination on gene expression, several classified genes were analyzed in receptacles by qRT-PCR (Figure 7B). Consistent with the transcriptome data, two *GH3* genes, four *GA20ox* genes, and *ACO1* gene were upregulated in response to both pollination and auxin, but more significantly induced under NAA treatment. *FvCKX1* expression was upregulated under NAA treatment while was repressed by pollination. *FvCKX6* and *FvCKX8* were not induced by auxin treatment and were inhibited by pollination.

Together with previous results, our work indicated that auxin can induce the rapid fruit growth and promote gene expression, including those functioned in phytohormone pathways, which is different from pollination, although pollination can stimulate auxin accumulation in achenes.

## Discussion

Fruit set can be induced by both pollination-dependent and -independent manners, and the dynamic hormone distribution plays key roles in fruit set (Fenn and Giovannoni, 2021; Sharif et al., 2022). Auxin, which is mostly accumulated in the fertilized achene, seems to play a central role in fruit set. Auxin coordinates with other hormones in regulating fruit set (Vriezen et al., 2008; Kumar et al., 2014). Gibberellin (GA) acts downstream of the auxin signal in promoting fruit growth (Kang et al., 2013; Lu et al., 2016; Shinozaki et al., 2020). In *F. vesca*, abscisic acid (ABA) represses fruit development initiation, while auxin suppresses ABA accumulation through upregulating the expression of *FvCYP707A4a* (Liao et al., 2018). In addition to GA and ABA, auxin undergoes crosstalk with other hormones, such as cytokinin, salicylic acid (SA), and ethylene (Carbonell-Bejerano et al., 2011; Marsch-Martinez et al., 2012; Ding et al., 2013; Shinozaki et al., 2018; Khew et al., 2020). Achenes and receptacles have different roles in fruit set initiation (McAtee et al., 2013). By testing the phytohormone contents, we found cytokinin, which was supposed to have a positive role in fruit set (Marsch-Martinez et al., 2012; Matsuo et al., 2012; Cucinotta et al., 2016; Aremu et al., 2020) was also accumulated in the fertilized achenes, while ABA and SA contents accumulated in receptacles at S1 and then downregulated after fertilization (Figure 1A, Supplementary Figure 1). Genes related to GA, cytokinin, ABA, and SA have different expression patterns after auxin application (Figure 7A). Taken together, we proposed a leading role of auxin during this process, and auxin produced in the fertilized achenes can directly (auxin signal transduction) or indirectly (influencing other hormone pathway, such as GA and ABA) initiate receptacle development to coordinate with the developing achenes.

Indeed, during normal fruit set, cell division and proliferation seemed to be the early events and controlled by auxin and GA (Fuentes and Vivian-Smith, 2009; Shakya and Lal, 2018). Studies suggested that fruit set was accomplished within 2–4 days after pollination by coordinating responses in fruit and seed (Kang et al., 2013; Shinozaki et al., 2020). Transcriptional regulation, signal transduction, and metabolism variation have already occurred in this phase (Kang et al., 2013; Liao et al., 2018; Shinozaki et al., 2020). However, the earliest stage of phytohormone accumulation, the coordination of phytohormones to regulate fruit development initiation, and the relationship between achenes and receptacles are largely enigmatic (Shinozaki et al., 2020). In this study, we found the promotion of pollination on enlargement of fruit happen as early as 1 DAA. Then the transcriptome analysis was performed in achenes and receptacles, respectively, to elucidate the molecular mechanism underlying fruit set initiation. To filter out the less sensitive genes and sift out some key genes, a high threshold [*p* < 0.05, |log2(fold change)| > 1.5] was set to define DEGs. From the transcriptome data, we found that auxin, but not pollination, was able to activate the expression of many growth-related genes, especially in receptacles, which resulted in the fast growth responses. Meanwhile, genes involved in other kinds of hormones, such as GA and cytokinin pathways, were also regulated by exogenous auxin treatment, but not pollination. Therefore, the early fruit growth induction of pollination occurred in an auxin-independent pathway. The process of auxin induced rapid fruit development happening in the lateral phase of fruit set (2–4 days after pollination).

Fruit development is to protect developing seeds and facilitate their dispersal (Di Marzo et al., 2020). In natural conditions, fruit development conformed with achene growth, and fleshy fruit development is repressed when seed are not fertilized until the fertilized achenes release auxin signals to the ovary wall and receptacles (Alabadi et al., 2009). Prior to auxin accumulation, genes related to cell wall metabolism, signal transduction and hormone synthesis have been differentially expressed in achene and receptacle were corresponding regulated to active cell wall metabolic, signal transduction, hormone biosynthesis and so on. Exogenous auxin mimics the auxin signal produced by fertilized achenes. To summarize the existing research, we re-draw the regulation mechanism of fruit set in strawberry (Figure 7C). Pollination activates cell wall metabolism and induces auxin accumulation in achenes, and then auxin is transported to receptacles and promotes fruit enlargement. Auxin induces GA accumulation both in achenes and receptacles to initiate cell division and proliferation processes on the one hand, and degrades ABA to cause growth inhibition in receptacles on the other hand. Auxin and GA may regulate achene growth in independent regulatory pathways but have a collaboration on receptacle development. This proposed working model needs to be verified by genetic analysis. Nonetheless, our findings provide a holistic insight into the molecular connection between achenes and receptacles during fruit set initiation and lay the foundation for genetic improvement of strawberry fruit, including parthenocarpic strawberry fruit.

## Data Availability Statement

The datasets presented in this study can be found in online repositories. The names of the repository/repositories and accession number(s) can be found in the article/Supplementary Material.

## Author Contributions

WT, TX, and YT initiated the projects and wrote the manuscript. YT conducted most of experiments. WX and XW contributed to plant sample preparation. JL helped with transcriptome annotation. JM and JH helped with the differentially expressed gene identification. All authors contributed to the article and approved the submitted version.

## Funding

This work was supported by National Natural Science Foundation of China (Grants 32130010 and 31870256) and internal funds from Fujian Agriculture and Forestry University to TX.

## Conflict of Interest

The authors declare that the research was conducted in the absence of any commercial or financial relationships that could be construed as a potential conflict of interest.

## Publisher's Note

All claims expressed in this article are solely those of the authors and do not necessarily represent those of their affiliated organizations, or those of the publisher, the editors and the reviewers. Any product that may be evaluated in this article, or claim that may be made by its manufacturer, is not guaranteed or endorsed by the publisher.

## Supplementary Material

Supplementary Figure 1 Changes of phytohormones contents in receptacle and achene during fruit development. The levels of zeatin **(A)**, ABA **(B)**, and SA **(C)** in achene and receptacle were measured. Error bars represent SD, *n* = 3. 15–20 fruits were used for each replicate. The significant difference between groups was labeled (*p* < 0.05, one way ANOVA).

Supplementary Figure 2 Fruits showed growth response 1 day after pollination. **(A)** The size of fruits and achenes with (Poll) and without pollination (No-poll). Dimension of fruits and achenes were characterized from−2 to 2 days after anthesis. Bar = 1 cm. **(B,C)** Vertical diameter and horizontal diameter quantification of fruits and achenes in **(A)**. Error bar in **(B)** showed the SD, *n* = 15–20. Error bars in **(C)** represent the SD, *n* = 60. Statistical analysis showed the significant difference between the groups (*p* < 0.05, one way ANOVA).

Supplementary Figure 3 Transcriptome analysis of achene and receptacle under different conditions. **(A)** Violin figure showed gene expression level for the identified unigenes. **(B)** Similariton for the classified groups basing on hole gene expression profile.

Supplementary Figure 4 The enriched GO terms identified in achene and receptacle under GA3 treatment. The up or down-regulated genes were noted in different color. Analysis was performed in receptacle and achene separately. *P* < 0.05, |log_2_(Fold change)| > 1.5.

Supplementary Figure 5 Statistical analysis of the DEGs identified in different treatment conditions. Numbers of the upregulated genes (orange) and downregulated genes (gray) were shown for each group. Poll represents pollination. *P* < 0.05, |log2(Fold change)| > 1.5.

Supplementary Figure 6 The DEGs specifically regulated by auxin treatment. **(A)** Expression pattern for genes significantly regulated by NAA treatment in receptacle or achene. **(B)** Functional enrichment of GO terms for genes special responding to NAA. *P* < 0.05, |log2(Fold change)| > 1.5.

Supplementary Table 1 The differentially expressed phytohormone pathways genes under pollination and NAA treatment comparing with no-pollination in achene.

Supplementary Table 2 The differentially expressed phytohormone pathways genes under pollination and NAA treatment comparing with no-pollination in receptacle.

Supplementary Table 3 Primers and accession IDs used for qRT-PCR.

## References

[B1] Alabadi D. Blazquez M. A. Carbonell J. Ferrandiz C. Perez-Amador M. A. (2009). Instructive roles for hormones in plant development. Int. J. Dev. Biol. 53, 1597–1608. 10.1387/ijdb.072423da 19247940

[B2] Aremu A. O. Fawole O. A. Makunga N. P. Masondo N. A. Moyo M. Buthelezi N. M. D.. (2020). Applications of cytokinins in horticultural fruit crops: trends and future prospects. Biomolecules 10:91222. 10.3390/biom10091222 32842660 PMC7563339

[B3] Cappelletti R. Sabbadini S. Mezzetti B. (2015). Strawberry (*Fragaria* x *ananassa*). Methods Mol. Biol. 1224, 217–227. 10.1007/978-1-4939-1658-0_18 25416261

[B4] Carbonell-Bejerano P. Urbez C. Granell A. Carbonell J. Perez-Amador M. A. (2011). Ethylene is involved in pistil fate by modulating the onset of ovule senescence and the GA-mediated fruit set in Arabidopsis. BMC Plant Biol. 11:84. 10.1186/1471-2229-11-84 21575215 PMC3124430

[B5] Carrera E. Ruiz-Rivero O. Peres L. E. Atares A. Garcia-Martinez J. L. (2012). Characterization of the procera tomato mutant shows novel functions of the SlDELLA protein in the control of flower morphology, cell division and expansion, and the auxin-signaling pathway during fruit-set and development. Plant Physiol. 160, 1581–1596. 10.1104/pp.112.204552 22942390 PMC3490602

[B6] Cucinotta M. Manrique S. Guazzotti A. Quadrelli N. E. Mendes M. A. Benkova E.. (2016). Cytokinin response factors integrate auxin and cytokinin pathways for female reproductive organ development. Development 143, 4419–4424. 10.1242/dev.143545 27737904

[B7] Di Marzo M. Herrera-Ubaldo H. Caporali E. Novak O. Strnad M. Balanza V.. (2020). SEEDSTICK controls arabidopsis fruit size by regulating cytokinin levels and FRUITFULL. Cell Rep. 30, 2846–2857 e2843. 10.1016/j.celrep.2020.01.101 32101756

[B8] Ding J. Chen B. Xia X. Mao W. Shi K. Zhou Y.. (2013). Cytokinin-induced parthenocarpic fruit development in tomato is partly dependent on enhanced gibberellin and auxin biosynthesis. PLoS ONE 8:e70080. 10.1371/journal.pone.0070080 23922914 PMC3726760

[B9] Dorcey E. Urbez C. Blazquez M. A. Carbonell J. Perez-Amador M. A. (2009). Fertilization-dependent auxin response in ovules triggers fruit development through the modulation of gibberellin metabolism in Arabidopsis. Plant J. 58, 318–332. 10.1111/j.1365-313X.2008.03781.x 19207215

[B10] Du L. Bao C. Hu T. Zhu Q. Hu H. He Q.. (2016). SmARF8, a transcription factor involved in parthenocarpy in eggplant. Mol. Genet. Genom. 291, 93–105. 10.1007/s00438-015-1088-5 26174736

[B11] Fenn M. A. Giovannoni J. J. (2021). Phytohormones in fruit development and maturation. Plant J. 105, 446–458. 10.1111/tpj.15112 33274492

[B12] Fuentes S. Ljung K. Sorefan K. Alvey E. Harberd N. P. Ostergaard L. (2012). Fruit growth in Arabidopsis occurs via DELLA-dependent and DELLA-independent gibberellin responses. Plant Cell. 24, 3982–3996. 10.1105/tpc.112.103192 23064323 PMC3517231

[B13] Fuentes S. Vivian-Smith A. (2009). Fertilisation and fruit initiation. Fruit Dev. Seed Dispersal 4, 107–171. 10.1002/9781444314557.ch4

[B14] Gillaspy G. Ben-David H. Gruissem. W. (1993). Fruits a developmental perspective. Plant Cell. 5, 1439–1451. 10.1105/tpc.5.10.1439 12271039 PMC160374

[B15] Giovannoni J. J. (2004). Genetic regulation of fruit development and ripening. Plant Cell. 16(Suppl.), S170–180. 10.1105/tpc.019158 15010516 PMC2643394

[B16] Goetz M. Vivian-Smith A. Johnson S. D. Koltunow A. M. (2006). AUXIN RESPONSE FACTOR8 is a negative regulator of fruit initiation in Arabidopsis. Plant Cell. 18, 1873–1886. 10.1105/tpc.105.037192 16829592 PMC1533983

[B17] Gorguet B. van Heusden A. W. Lindhout P. (2005). Parthenocarpic fruit development in tomato. Plant Biol. 7, 131–139. 10.1055/s-2005-837494 15822008

[B18] Gray W. M. Kepinski S. Rouse D. Leyser O. Estelle M. (2001). Auxin regulates SCF-dependent degradation of AUX IAA proteins. Nature 414, 271–276. 10.1038/35104500 11713520

[B19] Gu Q. C. F. Yanofsky F. M Martienssen R. (1998). The FRUITFULL MADS-box gene mediates cell differentiation during Arabidopsis fruit development. Development 125, 1509–1517. 10.1242/dev.125.8.1509 9502732

[B20] Handa A. K. Tiznado-Hernández M.-E. Mattoo A. K. (2012). Fruit development and ripening: a molecular perspective. Plant Biotechnol. Agri. 2, 405–424. 10.1016/B978-0-12-381466-1.00026-2

[B21] Hartl K. Denton A. Franz-Oberdorf K. Hoffmann T. Spornraft M. Usadel B.. (2017). Early metabolic and transcriptional variations in fruit of natural white-fruited *Fragaria vesca* genotypes. Sci Rep. 7:45113. 10.1038/srep45113 28327625 PMC5361166

[B22] Hollender C. A. Geretz A. C. Slovin J. P. Liu Z. (2012). Flower and early fruit development in a diploid strawberry, *Fragaria vesca*. Planta 235, 1123–1139. 10.1007/s00425-011-1562-1 22198460

[B23] Hu J. Israeli A. Ori N. Sun T. P. (2018). The interaction between DELLA and ARF/IAA mediates crosstalk between gibberellin and auxin signaling to control fruit initiation in tomato. Plant Cell. 30, 1710–1728. 10.1105/tpc.18.00363 30008445 PMC6139683

[B24] Ishibashi M. Yoshikawa H. Uno Y. (2017). Expression profiling of strawberry allergen Fra a during fruit ripening controlled by exogenous auxin. Int. J. Mol. Sci. 18:61186. 10.3390/ijms18061186 28574483 PMC5486009

[B25] Jong D. M. Mariani C. Vriezen W. H. (2009). The role of auxin and gibberellin in tomato fruit set. J. Exp. Bot. 60, 1523–1532. 10.1093/jxb/erp094 19321650

[B26] Kang C. Darwish O. Geretz A. Shahan R. Alkharouf N. Liu Z. (2013). Genome-scale transcriptomic insights into early-stage fruit development in woodland strawberry *Fragaria vesca*. Plant Cell. 25, 1960–1978. 10.1105/tpc.113.111732 23898027 PMC3723606

[B27] Khew C. Y. Mori I. C. Matsuura T. Hirayama T. Harikrishna J. A. Lau E. T.. (2020). Hormonal and transcriptional analyses of fruit development and ripening in different varieties of black pepper (*Piper nigrum*). J. Plant Res. 133, 73–94. 10.1007/s10265-019-01156-0 31853665

[B28] Kumar R. Khurana A. Sharma A. K. (2014). Role of plant hormones and their interplay in development and ripening of fleshy fruits. J. Exp. Bot. 65, 4561–4575. 10.1093/jxb/eru277 25028558

[B29] Liao X. Li M. Liu B. Yan M. Yu X. Zi H.. (2018). Interlinked regulatory loops of ABA catabolism and biosynthesis coordinate fruit growth and ripening in woodland strawberry. Proc. Natl. Acad. Sci. U. S. A. 115, E11542–E11550. 10.1073/pnas.1812575115 30455308 PMC6298082

[B30] Lu L. Liang J. Zhu X. Xiao K. Li T. Hu J. (2016). Auxin- and cytokinin-induced berries set in grapevine partly rely on enhanced gibberellin biosynthesis. Tree Genet. Genom. 12:4. 10.1007/s11295-016-0980-4

[B31] Marsch-Martinez N. Ramos-Cruz D. Irepan Reyes-Olalde J. Lozano-Sotomayor P. Zuniga-Mayo V. M. De Folter S. (2012). The role of cytokinin during *Arabidopsis gynoecia* and fruit morphogenesis and patterning. Plant J. 72, 222–234. 10.1111/j.1365-313X.2012.05062.x 22640521

[B32] Marti C. Orzaez D. Ellul P. Moreno V. Carbonell J. Granell A. (2007). Silencing of DELLA induces facultative parthenocarpy in tomato fruits. Plant J. 52, 865–876. 10.1111/j.1365-313X.2007.03282.x 17883372

[B33] Matsuo S. Kikuchi K. Fukuda M. Honda I. Imanishi S. (2012). Roles and regulation of cytokinins in tomato fruit development. J. Exp. Bot. 63, 5569–5579. 10.1093/jxb/ers207 22865911 PMC3444270

[B34] McAtee P. Karim S. Schaffer R. David K. (2013). A dynamic interplay between phytohormones is required for fruit development, maturation, and ripening. Front. Plant Sci. 4:79. 10.3389/fpls.2013.00079 23616786 PMC3628358

[B35] Nir I. Shohat H. Panizel I. Olszewski N. Aharoni A. Weiss D. (2017). The tomato DELLA protein PROCERA acts in guard cells to promote stomatal closure. Plant Cell. 29, 3186–3197. 10.1105/tpc.17.00542 29150547 PMC5757276

[B36] Nitsch J. P. (1950). Growth and morphogenesis of the strawberry as related to auxin. Am. J. Bot. 37, 211–215. 10.1002/j.1537-2197.1950.tb12183.x25855820

[B37] Ohad N. Margossian L. Hsu Y. Williams C. Repetti P. Fischer R. L. (1996). A mutation that allows endosperm development without fertilization. Plant Biol. 93, 5319–5324. 10.1073/pnas.93.11.5319 11607683 PMC39243

[B38] Radke J. Ishaque N. Koll R. Gu Z. Schumann E. Sieverling L.. (2022). The genomic and transcriptional landscape of primary central nervous system lymphoma. Nat. Commun. 13:2558. 10.1038/s41467-022-30050-y 35538064 PMC9091224

[B39] Roeder A. H. Yanofsky M. F. (2006). Fruit development in Arabidopsis. Arabidopsis Book 4:e0075. 10.1199/tab.0075 22303227 PMC3243326

[B40] Serrani J. C. Fos M. Atarés A. García-Martínez J. (2007). Effect of gibberellin and auxin on parthenocarpic fruit growth induction in the cv micro-tom of tomato. J. Plant Growth Regul. 26, 211–221. 10.1007/s00344-007-9014-7

[B41] Serrani J. C. Ruiz-Rivero O. Fos M. Garcia-Martinez J. L. (2008). Auxin-induced fruit-set in tomato is mediated in part by gibberellins. Plant J. 56, 922–934. 10.1111/j.1365-313X.2008.03654.x 18702668

[B42] Shakya R. Lal M. A. (2018). Fruit development and ripening. Plant Physiol. Dev. Metabol. 27, 857–883. 10.1007/978-981-13-2023-1_27

[B43] Sharif R. Su L. Chen X. Qi X. (2022). Hormonal interactions underlying parthenocarpic fruit formation in horticultural crops. Hortic Res. 9:uhab024. 10.1093/hr/uhab024 35031797 PMC8788353

[B44] Shinozaki Y. Beauvoit B. P. Takahara M. Hao S. Ezura K. Andrieu M. H.. (2020). Fruit setting rewires central metabolism *via* gibberellin cascades. Proc. Natl. Acad. Sci. U. S. A. 117, 23970–23981. 10.1073/pnas.2011859117 32883877 PMC7519230

[B45] Shinozaki Y. Ezura H. Ariizumi T. (2018). The role of ethylene in the regulation of ovary senescence and fruit set in tomato (*Solanum lycopersicum*). Plant Signal Behav. 13:e1146844. 10.1080/15592324.2016.1146844 26934126 PMC5933915

[B46] Slovin J. P. Schmitt K. Folta K. M. (2009). An inbred line of the diploid strawberry (*Fragaria vesca* f.) semperflorens for genomic and molecular genetic studies in the Rosaceae. Plant Methods 5:15. 10.1186/1746-4811-5-15 19878589 PMC2780397

[B47] Su L. Rahat S. Ren N. Kojima M. Qi X. (2021). Cytokinin and auxin modulate cucumber parthenocarpy fruit development. Scientia Horticult. 282:26. 10.1016/j.scienta.2021.110026

[B48] Sun T. P. (2011). The molecular mechanism and evolution of the GA-GID1-DELLA signaling module in plants. Curr Biol. 21, R338–R345. 10.1016/j.cub.2011.02.036 21549956

[B49] Viviansmith A. Luo M. Chaudhury A. Koltunow A. (2001). Fruit development is actively restricted in the absence of fertilization in Arabidopsiss. Development 128, 2321–2331. 10.1242/dev.128.12.2321 11493551

[B50] Vriezen W. H. Feron R. Maretto F. Keijman J. Mariani C. (2008). Changes in tomato ovary transcriptome demonstrate complex hormonal regulation of fruit set. New Phytol. 177, 60–76. 10.1111/j.1469-8137.2007.02254.x 18028300

[B51] Wang H. Jones B. Li Z. Frasse P. Delalande C. Regad F.. (2005). The tomato Aux/IAA transcription factor IAA9 is involved in fruit development and leaf morphogenesis. Plant Cell. 17, 2676–2692. 10.1105/tpc.105.033415 16126837 PMC1242265

[B52] Yang L. Smyth G. K. Wei S. (2014). Feature counts an efficient general-purpose program for assigning sequence reads to genomic features. Bioinformatics. 7, 923–930. 10.1093/bioinformatics/btt656 24227677

[B53] Zhang J. Chen R. Xiao J. Qian C. Wang T. Li H.. (2007). A single-base deletion mutation in SlIAA9 gene causes tomato (*Solanum lycopersicum*) entire mutant. J. Plant Res. 120, 671–678. 10.1007/s10265-007-0109-9 17955175

[B54] Zhou J. Sittmann J. Guo L. Xiao Y. Huang X. Pulapaka A.. (2021). Gibberellin and auxin signaling genes RGA1 and ARF8 repress accessory fruit initiation in diploid strawberry. Plant Physiol. 185, 1059–1075. 10.1093/plphys/kiaa087 33793929 PMC8133647

