# Supplementary material for: Auxin Coordinates Achene and Receptacle Development During Fruit Initiation in Fragaria vesca
Source: Front Plant Sci. 2022 Jul 7;13:929831. doi: 10.3389/fpls.2022.929831 (PMC9301465; doi:10.3389/fpls.2022.929831)
Supplement: Supplementary Figure 1 — Changes of phytohormones contents in receptacle and achene during fruit development. The levels of zeatin (A), ABA (B), and SA (C) in achene and receptacle were measured. Error bars represent SD, n = 3. 15–20 fruits were used for each replicate. The significant difference between groups was labeled (p < 0.05, one way ANOVA). [file Data_Sheet_1.docx]

Supplementary Material

# Supplementary Figures and Tables

## Supplementary Figures


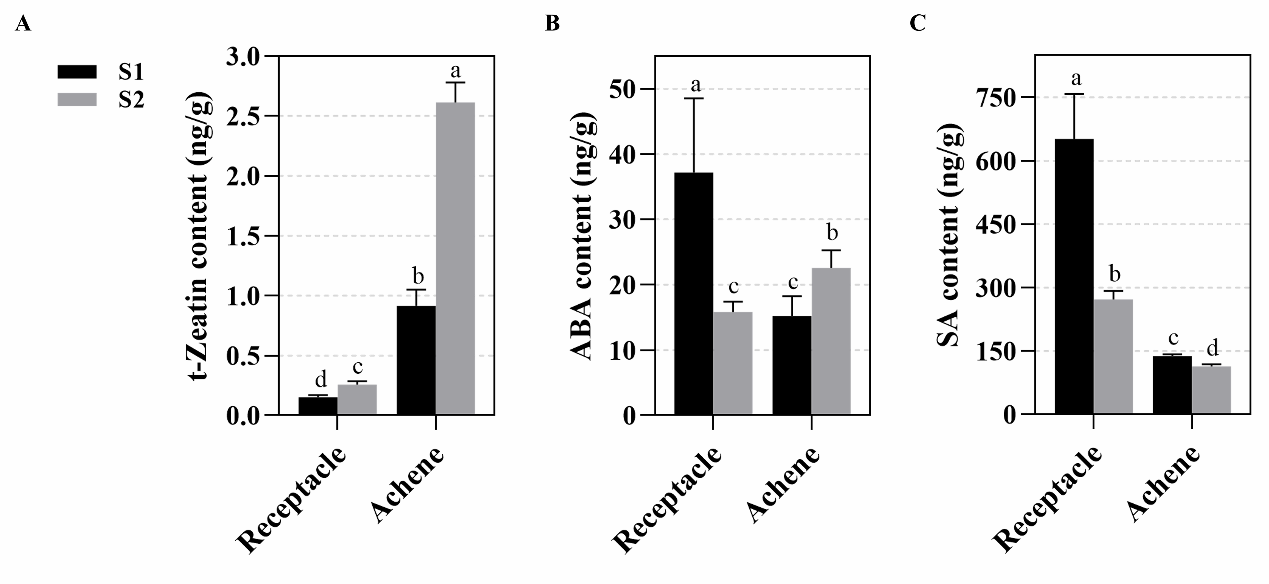


**SUPPLEMENTARY FIGURE S1 |** Changes of phytohormones contents in receptacle and achene during fruit development. The levels of zeatin (**A**), ABA (**B**) and SA (**C**) in achene and receptacle were measured. Error bars represent SD, n = 3. 15 ~ 20 fruits were used for each replicate. The significant different between groups was labeled (*p* < 0.05, one way ANOVA).


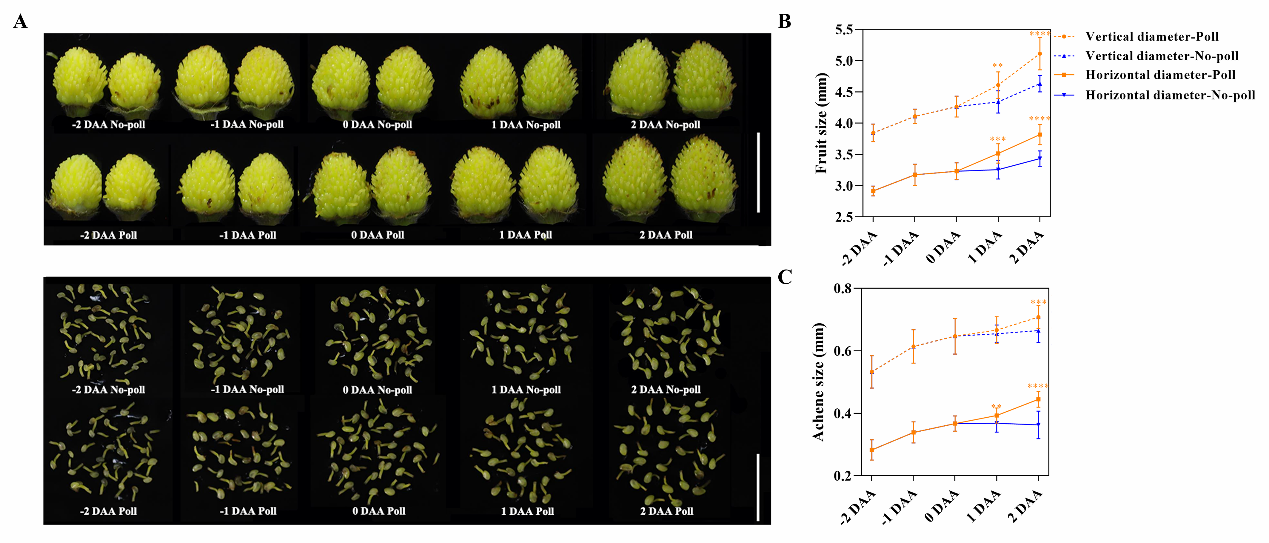


**SUPPLEMENTARY FIGURE S2 |** Fruits showed growth response 1 day after pollination. (**A**) The size of fruits and achenes with (Poll) and without pollination (No-poll). Dimension of fruits and achenes were characterized from -2 to 2 days after anthesis. Bar = 1 cm. (**B** and **C**) Vertical diameter and horizontal diameter quantification of fruits and achenes in **A**. Error bar in (**B)** showed the SD, n = 15 - 20. Error bars in (**C**) represent the SD, n = 60. Statistical analysis showed the significant difference between the groups (*p* < 0.05, one way ANOVA).


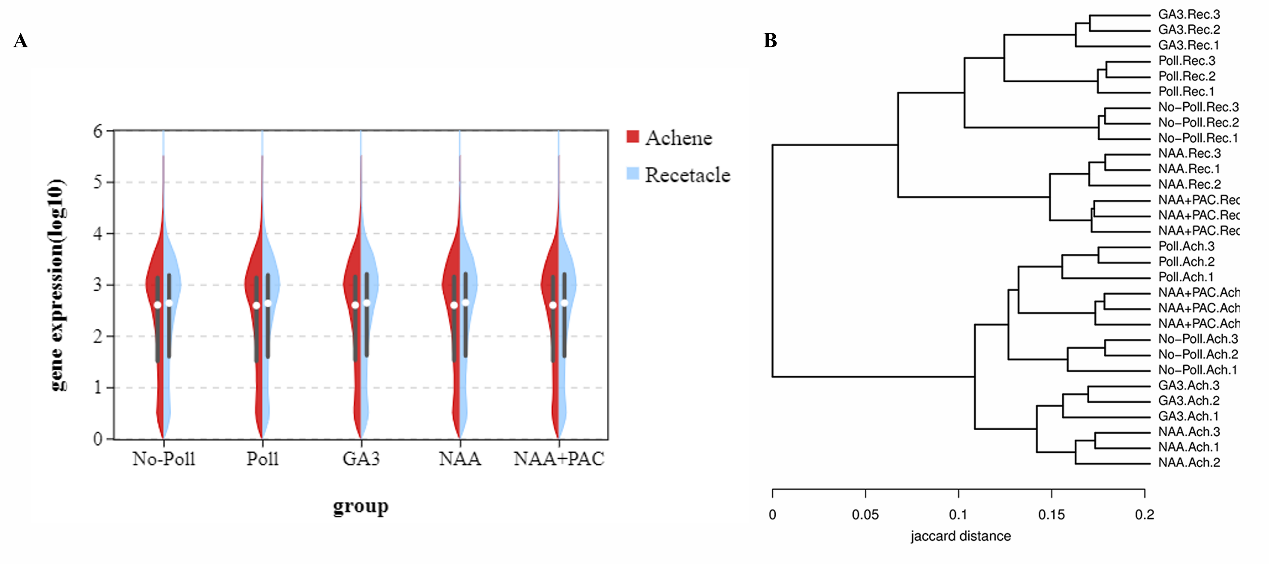


**SUPPLEMENTARY FIGURE S3 |** Transcriptome analysis of achene and receptacle under different conditions. (**A**) Violin figure showed gene expression level for the identified unigenes. (**B**) Similariton for the classified groups basing on hole gene expression profile.


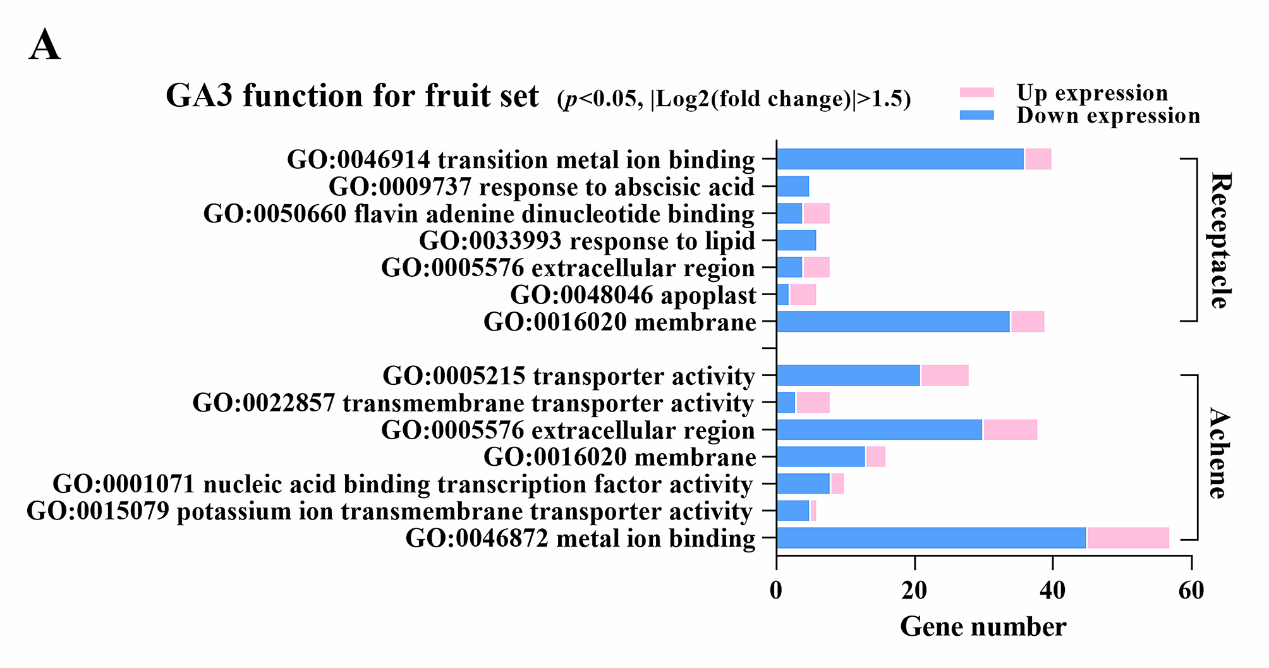


**SUPPLEMENTARY FIGURE S4 |** The enriched GO terms identified in achene and receptacle under GA3 treatment. The up or down-regulated genes were noted in different color. Analysis was performed in receptacle and achene separately. *p* < 0.05, |log_2_(Fold change)| > 1.5.


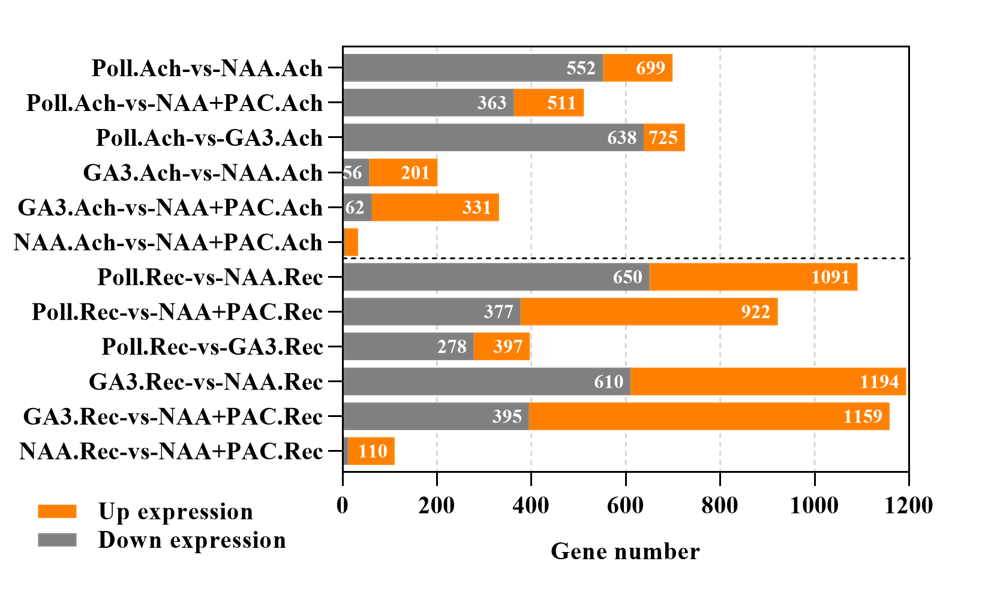


**SUPPLEMENTARY FIGURE S5 |** Statistical analysis of the DEGs identified in different treatment conditions. Numbers of the up-regulated genes (orange) and down-regulated genes (gray) were shown for each group. Poll represents pollination. *p* < 0.05, |log2(Fold change)| > 1.5.
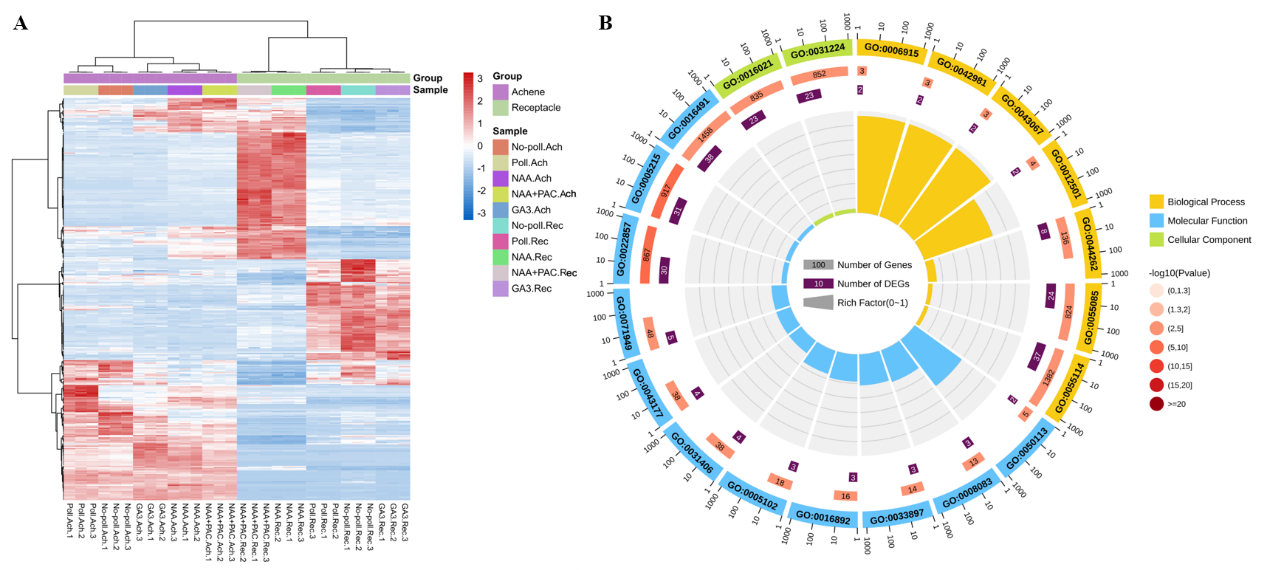


**SUPPLEMENTARY FIGURE S6 |** The DEGs specifically regulated by auxin treatment. (**A**) Expression pattern for genes significantly regulated by NAA treatment in receptacle or achene. (**B**) Functional enrichment of GO terms for genes special responding to NAA. *p* < 0.05, |log2(Fold change)| > 1.5.

## Supplementary Tables

**Supplementary Table S1.** The differentially expressed phytohormone pathways genes under pollination and NAA treatment comparing with no-pollination in achene.

| **Pathway** | **Gene ID** | **Description (Ab)** | **No-Poll.Ach-vs-Poll.Ach log2(FC)** | **No-Poll.Ach-vs-NAA.Ach**  **log2(FC)** |
| --- | --- | --- | --- | --- |
| uxin pathway | FvH4_2g04750 | GH3.1, putative indole-3-acetic acid-amido synthetase GH3.1 | 2.6 | 4.9 |
|  | FvH4_4g22430 | GH3.17, Indole-3-acetic acid-amido synthetase | - | 3.1 |
|  | FvH4_1g16980 | GH3.2, indole-3-acetic acid-amido synthetase 3.2 | 2.3 | 5.8 |
|  | FvH4_2g25330 | GH3.9, indole-3-acetic acid-amido synthetase 3. | - | 3.9 |
|  | FvH4_2g20500 | IAA12, indoleacetic acid-induced protein 12 | - | 1.8 |
|  | FvH4_2g35280 | IAA26a, indoleacetic acid-induced protein 26a | - | 1.6 |
|  | FvH4_6g30860 | IAA6, indoleacetic acid-induced protein 6 | - | 3.6 |
|  | FvH4_3g23570 | IAMT1, Indole-3-acetate O-methyltransferase 1 | - | 2.6 |
|  | FvH4_5g22780 | SAUR-like auxin-responsive protein family | - | 2.1 |
| Gibberellin biosynthesis | FvH4_7g28670 | GA20OX1, Gibberellin 20 oxidase 1 | - | 1.8 |
|  | FvH4_3g16760 | GA2OX1, gibberellin 2-oxidase 1 | - | -3 |
|  | FvH4_4g36510 | GA2OX8, Gibberellin 2-beta-dioxygenase 8 | 3.1 | 2.7 |
|  | FvH4_6g30780 | GA3OX1, Gibberellin 3-beta-dioxygenase 1 | - | - |
|  | FvH4_2g24370 | GID1B, Gibberellin receptor GID1B | - | -2 |
|  | FvH4_2g33690 | GID2, F-box protein GID2 | - | -2 |
| Cytokinin biosynthesis | FvH4_1g07610 | CKX5, Cytokinin dehydrogenase 5 | 1.6 | - |
|  | FvH4_4g14440 | IPT; adenylate dimethylallyltransferase (cytokinin synthase) | - | -3 |
|  | FvH4_6g17030 | UDP-glycosyltransferase 73C1-like | - | -2 |
| Ethylene biosynthesis | FvH4_1g07181 | ACO1, 1-aminocyclopropane-1-carboxylate oxidase 1 | - | 1.9 |
|  | FvH4_7g15310 | ACS1, 1-aminocyclopropane-1-carboxylate synthase 1 | 3.8 | 2.3 |
|  | FvH4_7g09550 | ERF034, Ethylene-responsive transcription factor 034 | - | -2 |
| Abscisic acid biosynthesis | FvH4_5g25150 | CYP707A2, Abscisic acid 8'-hydroxylase | 1.7 | - |
|  | FvH4_3g16730 | NCED1, 9-cis-epoxycarotenoid dioxygenase, chloroplastic | 2.5 | - |

**Supplementary Table S2.** The differentially expressed phytohormone pathways genes under pollination and NAA treatment comparing with no-pollination in receptacle.

| **Pathway** | **Gene ID** | **Description (Ab)** | **No-Poll.Rec-vs-Poll.Rec log2(FC)** | **No-Poll.Rec-vs-NAA. Rec log2(FC)** |
| --- | --- | --- | --- | --- |
| Auxin pathway | FvH4_2g38760 | ARF5, Auxin response factor 5 | - | -1.7 |
|  | FvH4_2g02300 | ARG7, Indole-3-acetic acid-induced protein 7 | - | 1.52 |
|  | FvH4_5g22690 | ARG7, Indole-3-acetic acid-induced protein 7 | - | 1.99 |
|  | FvH4_5g22700 | Auxin-induced protein 15A | - | 1.67 |
|  | FvH4_2g04750 | GH3.1, putative indole-3-acetic acid-amido synthetase GH3.1 | - | 5.8 |
|  | FvH4_4g22430 | GH3.17, Indole-3-acetic acid-amido synthetase | 1.76 | 5.65 |
|  | FvH4_1g16980 | GH3.2, indole-3-acetic acid-amido synthetase 3.2 | - | 6.29 |
|  | FvH4_2g25330 | GH3.9, indole-3-acetic acid-amido synthetase 3. | - | 4.49 |
|  | FvH4_2g20500 | IAA12, indoleacetic acid-induced protein 12 | - | 2.75 |
|  | FvH4_2g22520 | IAA14b, indoleacetic acid-induced protein 14b | - | 3.14 |
|  | FvH4_6g30850 | IAA17, indoleacetic acid-induced protein 17 | - | 2.89 |
|  | FvH4_2g22530 | IAA4b, indoleacetic acid-induced protein 4b | - | 2.31 |
|  | FvH4_6g30860 | IAA6, indoleacetic acid-induced protein 6 | - | 4.92 |
|  | FvH4_2g27160 | IAA-amino acid hydrolase ILR1-like 6 | - | -1.5 |
|  | FvH4_3g23570 | IAMT1, Indole-3-acetate O-methyltransferase 1 | - | 4.09 |
|  | FvH4_5g17310 | PIN1, Auxin efflux carrier component 1 | - | 2.48 |
|  | FvH4_2g02310 | SAUR23, Auxin-responsive protein 23 | - | 1.64 |
|  | FvH4_7g17340 | SAUR32, Auxin-responsive protein 32 | - | 1.5 |
|  | FvH4_7g11280 | SAUR36, Auxin-responsive protein 36 | - | -2.2 |
|  | FvH4_5g22780 | SAUR-like auxin-responsive protein family | - | 2.25 |
|  | FvH4_5g22810 | SAUR-like auxin-responsive protein family | - | 4.11 |
| Gibberellin pathway | FvH4_7g28670 | GA20OX1, Gibberellin 20 oxidase 1 | - | 4.08 |
|  | FvH4_7g12600 | GA20OX2, Gibberellin 20 oxidase 2 | - | 5.01 |
|  | FvH4_3g16760 | GA2OX1, gibberellin 2-oxidase 1 | -3 | -6.7 |
|  | FvH4_3g05530 | GA2OX2, Gibberellin 2-beta-dioxygenase 2 | - | -1.6 |
|  | FvH4_3g38920 | GA2OX9, Gibberellin 2-oxidase 9 | - | -2.3 |
|  | FvH4_6g30780 | GA3OX1, Gibberellin 3-beta-dioxygenase 1 | - | 2.99 |
|  | FvH4_5g14950 | Gibberellin-regulated protein 6 | - | 6.33 |
|  | FvH4_3g11720 | GID1; gibberellin receptor GID1 | - | -4 |
|  | FvH4_6g04960 | GID1; gibberellin receptor GID1 | - | -2.1 |
|  | FvH4_2g24370 | GID1B, Gibberellin receptor GID1B | - | -2.4 |
|  | FvH4_2g33690 | GID2, F-box protein GID2 | - | -1.9 |
| Cytokinin pathway | FvH4_6g04970 | AHP1, Histidine-containing phosphotransferase protein 1 | - | -2.7 |
|  | FvH4_3g03260 | CKX; cytokinin dehydrogenase | - | 2.39 |
|  | FvH4_7g02150 | CKX1, Cytokinin dehydrogenase 1 | - | 2.66 |
|  | FvH4_2g23840 | IPT; adenylate dimethylallyltransferase (cytokinin synthase) | - | -4.8 |
|  | FvH4_4g14440 | IPT; adenylate dimethylallyl transferase (cytokinin synthase) | -1.5 | -6.6 |
|  | FvH4_3g31870 | LOG1, Cytokinin riboside 5'-monophosphate phosphoribohydrolase 1 | - | 3.8 |
|  | FvH4_6g16020 | LOG3, Cytokinin riboside 5'-monophosphate phosphoribohydrol | - | 3.24 |
|  | FvH4_3g04550 | LOG7, Cytokinin riboside 5'-monophosphate phosphoribohydrolase 7 | - | -2.7 |
|  | FvH4_2g31610 | LOG8, Cytokinin riboside 5'-monophosphate phosphoribohydrolase 8 | - | -1.8 |
| Ethylene pathway | FvH4_1g07181 | ACO1, 1-aminocyclopropane-1-carboxylate oxidase 1 | - | 1.7 |
|  | FvH4_3g01280 | ACO2, 1-aminocyclopropane-1-carboxylate oxidase 2 | - | -3 |
|  | FvH4_6g02270 | Ethylene-responsive transcription factor 1B | 2.23 | 5.4 |
|  | FvH4_6g08370 | SAMS1, S-adenosylmethionine synthase 1 | - | 1.51 |
|  | FvH4_4g21340 | SAMS2, S-adenosylmethionine synthase 2 | - | 2.51 |
| Abscisic acid pathway | FvH4_1g08010 | PP2C16, Protein phosphatase 2C 16 | - | 1.56 |
|  | FvH4_7g31810 | PP2C8, Probable protein phosphatase 2C 8 | -1.5 | -2.9 |
|  | FvH4_1g20310 | SNRK2; serine/threonine-protein kinase SRK2 | - | -2.3 |

**Supplementary Table S3.** Primers and Accession IDs used for qRT-PCR.

| Gene name | Forward primer (5'-3') | Reverse primer (5'-3') | Gene ID |
| --- | --- | --- | --- |
| FvACTIN | CAGAAAGATGCTTATGTCGG | TGGGGCAACACGAAGCTCAT |  |
| FvGH3.17 | CGAGCGAATTGCCAATGGAG | GCCACCAAACCAGAAGGAGT | FvH4_4g22430 |
| FvGH3.9 | CCTCGAACGAAACTGGGTCA | GATTTGCGGCAATCAGGGTC | FvH4_2g25330 |
| FvGA20ox1 | GCAATGGCAGTTGAGTGTATG | TGTTTGGGTATGTCAGTTTCGT | FvH4_7g28670 |
| FvGA20ox2 | GGCAGGTTCTCCTCCAAACT | TCTTCACCCATCACATTCACG | FvH4_7g12600 |
| FvGA20ox4 | GTCCCTGCCCTATTGACTCTT | TGGCAGCATTCTCGGTTG | FvH4_2g35050 |
| FvGA20ox5 | GGCTTGGACTAATGGGAGG | TTGTGGCAGAATCAGAGGG | FvH4_5g19970 |
| FvACO1 | GCGTCATCCTTCTGTTCCA | GCCATCTGTCTGGGCAATC | FvH4_6g42090 |
| FvCKX1 | CCACACCCATGGCTCAATCT | TAGGGCCGTTGCTTGTTTCT | FvH4_7g02150 |
| FvCKX6 | AGCTTGAAGTCGTTACGGGG | TCGAGCTCTGGTGATGATGC | FvH4_2g30990 |
| FvCKX8 | CTGGGAAACAAAACCAGCGG | CCGACGGTTCTGATTGGTGA | FvH4_2g39230 |
